# Consensus Statement on Managing Anxiety and Depression in Individuals with Inflammatory Bowel Disease

**DOI:** 10.1093/ibd/izae151

**Published:** 2024-08-22

**Authors:** Laurie Hinnant, Nicholas Rios Villacorta, Eliza Chen, Donna Bacchus, Jennifer Dotson, Ruby Greywoode, Laurie Keefer, Stephen Lupe, Leah Maggs, Garrett Meek, Eva Szigethy, Kathryn Tomasino, Orna G Ehrlich, Sylvia Ehle

**Affiliations:** Health Practice Area, RTI International, Research Triangle Park, NC, USA; Health Practice Area, RTI International, Research Triangle Park, NC, USA; Health Practice Area, RTI International, Research Triangle Park, NC, USA; College of Nursing and Health Innovation, University of Texas at Arlington, Arlington, TX, USA; Division of Gastroenterology, Hepatology and Nutrition, The Research Institute, Nationwide Children’s Hospital, Columbus, OH, USA; Department of Pediatrics, The Ohio State University College of Medicine, Columbus, OH, USA; Division of Gastroenterology, Montefiore Medical Center, Bronx, NY, USA; Icahn School of Medicine, Mount Sinai, New York, NY, USA; Department of Gastroenterology, Hepatology & Nutrition, Cleveland Clinic, Cleveland, OH, USA; Patient Advocate, Seattle, WA, USA; Patient Advocate, Boulder, CO, USA; Pediatric Psychiatry, Akron Children’s Hospital, Akron, OH, USA; Department of Pediatrics, University of Pittsburgh Medical Center, Pittsburgh, PA, USA; Feinberg School of Medicine, Northwestern University, Chicago, IL, USA; National Headquarters, Crohn’s & Colitis Foundation, New York, NY, USA; National Headquarters, Crohn’s & Colitis Foundation, New York, NY, USA

**Keywords:** inflammatory bowel disease, IBD, anxiety, depression, screening, treatment

## Abstract

**Background:**

Studies have found a higher risk of comorbid anxiety and depression among patients with inflammatory bowel disease (IBD) compared with healthy individuals. If left untreated, comorbid depression and anxiety in patients with IBD can lead to poorer health outcomes and an increased healthcare utilization. The goal of this work was to develop a consensus statement to begin to address patient and provider needs and responsibilities related to screening and treatment of depression and anxiety symptoms among patients with IBD.

**Methods:**

A literature scan was conducted to gather evidence-based background information and recommendations on the screening, diagnosis, and treatment of anxiety and depression in patients with IBD. This was followed by the engagement of a panel of IBD and mental health experts and patient advocates using a modified Delphi process to synthesize the literature and distill the information into a core set of statements to support provider actions and care delivery.

**Results:**

Six statements were distilled from the literature and consensus process that link to the general management, screening, and treatment of anxiety and depression in patients with IBD.

**Conclusions:**

Mental healthcare and support for IBD patients is critical; the statements included in this article represent practical considerations for IBD healthcare professionals in addressing key issues on provider awareness, knowledge and behaviors, screening and treatment resources, and patient education.

## Graphical Abstract

**Graphical Abstract:**
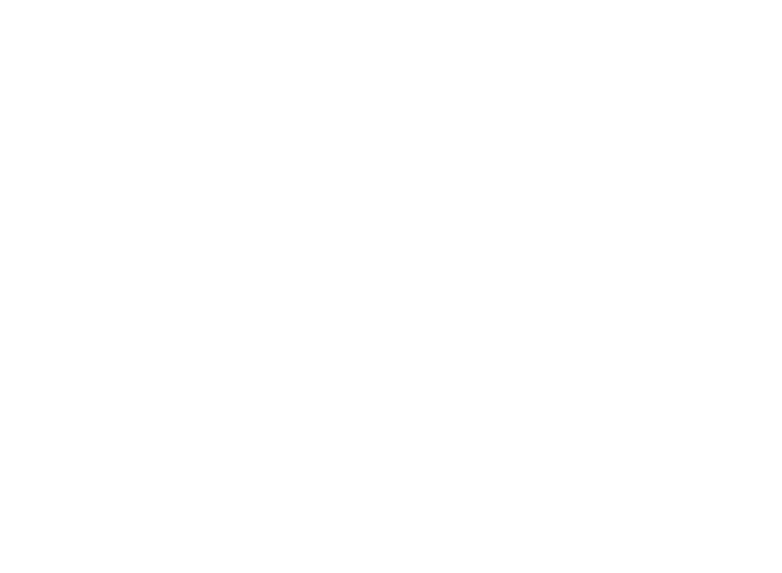

**Key Messages**

**What is already known?**

- People with inflammatory bowel disease (IBD) are more likely to experience symptoms of anxiety and depression than similar healthy individuals and need support to manage those symptoms as a part of routine IBD care.

**What is new here?**

- This article draws from the literature, provider, and patient experiences to communicate 6 practical consensus statements to support IBD professionals and patients around screening and treatment of anxiety and depression symptoms.

**How can this study help patient care?**

- This study seeks to raise IBD healthcare provider awareness of and resources for practical strategies they can adopt to improve patient care delivery around anxiety and depression.

## Introduction

Inflammatory bowel diseases (IBDs), such as ulcerative colitis and Crohn’s disease, are chronic immune-mediated diseases that cause inflammation throughout the gastrointestinal tract.^1^ Nearly 1 in 100 Americans are diagnosed IBD.^2^

Although IBD is a gastrointestinal disease, it can have a profound impact on a patient’s emotional health. Studies have found a higher prevalence of psychiatric comorbidities, such as anxiety and depression, among patients with IBD. Compared with similar healthy individuals, patients with IBD were found to have 3 to 5 times higher risk of developing anxiety disorders and 2 to 4 times higher risk of developing depression disorders in their lifetime.^3,4^ These psychiatric comorbidities can have significant implications for IBD patients, with evidence supporting an association between psychiatric state and IBD disease course, such as patients with more depressive symptoms presenting with greater inflammation due to stressors.^5^ Depressive symptoms are also associated with IBD relapse and shorter periods of remission.^6^ Evidence suggests that this may be due to the impact of psychological stress on neuroenteric pathways.^7^

Research also indicates a bidirectional relationship between IBD disease status and psychiatric comorbidities.^8-10^ Patients with depressive symptoms are at an increased risk of having and developing gastrointestinal disorders. Furthermore, there is evidence of an association between depressive state and subsequent deterioration in disease course of Crohn’s disease.^1^ This bidirectional relationship is worth exploring through the lens of a healthcare provider, as there may be an intersection between screening and treatment practices for IBD patients and patients with anxiety and depression. It is suggested that patients with IBD should be monitored for psychological well-being.^11^

Exploring the screening and treatment practices currently available and commonly practiced is important due to the impact psychiatric comorbidities may have on IBD care. IBD is among the costliest chronic conditions, particularly among patients with both IBD and mental health diseases.^12^ If left untreated, comorbid depression and anxiety in patients with IBD can lead to poorer health outcomes and increased healthcare utilization.^13^ Addressing anxiety and depression were identified as 2 potential areas of focus to prevent hospital readmission in patients with IBD.^14^ The integration of psychological support into routine IBD care was found to be important to holistic patient care of this population. Additionally, patients offered this care were found to be receptive to it, and the health outcomes were found to be positive.^15^

The goal of this work was to develop a consensus statement to begin to address patient and provider needs related to screening and treatment of depression and anxiety symptoms among patients with IBD. This was accomplished via a literature scan to gather evidence-based background information and recommendations, followed by the engagement of a panel of IBD and mental health experts using a modified Delphi process^16^ to synthesize the literature and distill the information into a core set of statements to support provider actions and care delivery.

## Methods

### Literature Scan

We conducted a literature scan using multiple electronic databases, including PubMed, Web of Science, and APA PsycInfo, to identify articles published between 2012 and 2023. In addition, a hand search of the Crohn’s and Colitis Foundation’s *Crohn’s & Colitis 360* online journal was conducted. Search terms included “Crohn’s,” “Colitis,” “inflammatory bowel disease,” “ulcerative colitis,” “depression,” “depressive disorder,” “anxiety,” “mental health,” “mental disorder,” “psychiatric illness,” “psychological wellbeing,” “systematic review,” “meta-analysis,” “randomized control trial,” “screening,” “diagnosis,” and “therapies.” These terms were used to generate an initial list of publications to be searched.

Studies were selected for inclusion if they discussed practical applications of screening, diagnosis, and treatment approaches for patients diagnosed with IBD alongside symptomatic or comorbid anxiety and/or depression. Only studies reported in the English language and conducted in countries designated as very highly developed per the United Nations Human Development Index were included.^17,18^ Research including psychiatric illnesses other than anxiety or depression were excluded, as they were considered separate entities from the priority area of focus.

Two reviewers (N.R.V. and E.C.) independently screened the complete list of publications between 2012 and August 2023. The resulting titles and abstracts were screened for inclusion into the review against the predetermined inclusion and exclusion criteria. A third reviewer was used to resolve any discrepancies by discussion. The original articles were retrieved for retained abstracts, and full-text reviews of the articles were conducted to further assess eligibility. Relevant data from the final list of included research articles were extracted into an article summary matrix, including study design, population size and characteristics, and key factors related to the screening, diagnosis, and treatment of anxiety and depression in patients with IBD.

### Elicitation Process

A panel of 9 experts in IBD-related care was engaged to review the literature scan and provide input as a part of the elicitation process. Panelist backgrounds included IBD healthcare clinicians, mental health professionals with expertise in working with patients with IBD, and IBD patient advocates. The goal of the panel was to synthesize relevant information from the literature as well as patient and provider experiences to develop a set of statements to help IBD healthcare professionals navigate patients’ needs related to anxiety and depression. Panelists were engaged in a modified Delphi process that included a review of background materials identified through the literature scan, followed by 2 virtual meetings and 3 asynchronous sessions of panel review and comment. The modified Delphi process was selected because it allowed for input from a wide variety of experts, much of the input could be collected anonymously to encourage honest dialogue and feedback, and while it was a more lengthy process, it included multiple opportunities to revisit decisions made at each step to ensure that there was broad agreement on the final statements. Engagement was conducted using a combination of the XLeap virtual engagement system and Zoom. XLeap allows for real-time and asynchronous engagement during the elicitation process in which panelists can see and respond to feedback from other members.

**Figure 1.**
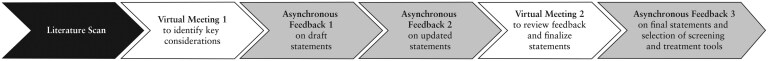
Modified Delphi process,

Materials were created to synthesize the key findings from the literature scan, including recommendations from study authors and a compilation of depression and anxiety screening and treatment approaches. These materials were shared with panel members in advance of the first virtual meeting. The 5-step modified Delphi process is described subsequently and in Figure 1. The first virtual meeting was used to gather input on key considerations in developing the consensus statements, including the role of IBD healthcare professionals in providing screening and treatment to patients around anxiety and depression, and key issues related to screening and treatment of anxiety and depression in individuals with IBD. Feedback was obtained from 8 of the 9 panel members at this meeting, and this feedback was used to develop an initial set of draft consensus statements that were shared with panelists for asynchronous review and scoring via XLeap. During the second round, all 9 panelists submitted qualitative feedback and reflected on draft language for the consensus statements. The third round consisted of panelist review and feedback via XLeap on the language for the updated consensus statements that incorporated the feedback provided during the prior round. All 9 panelists provided qualitative feedback on the language of the statements and conducted a rank-order exercise to prioritize statements within the 3 categories of general management, screening, and treatment. Panelists were also encouraged to provide qualitative feedback on their ratings. The mean score and normalized standard deviation for each statement were calculated using XLeap.^19^ The normalized standard deviation is an indication of consensus within the group in which below 0.2 indicates general to strong consensus in the group and above 0.3 indicates strong dissent. Two initial draft statements obtained a normalized standard deviation of 0.32, the only statements above the 0.3 threshold for consensus. In these cases, qualitative feedback was used to clarify the statements and, in both cases, these statements were combined with other statements as the primary concern was duplication of other statements. The study team utilized the qualitative feedback and the ratings to create a final set of statements for panel review. The fourth round brought participants together through a virtual meeting to reflect on the previous comments, review feedback, and finalize language for the consensus statements. The final round was conducted asynchronously, and all 9 participants provided feedback on the final statements and input on the final screening tools and treatment approaches to be highlighted.

## Results

### Literature Scan

**Figure 2.**
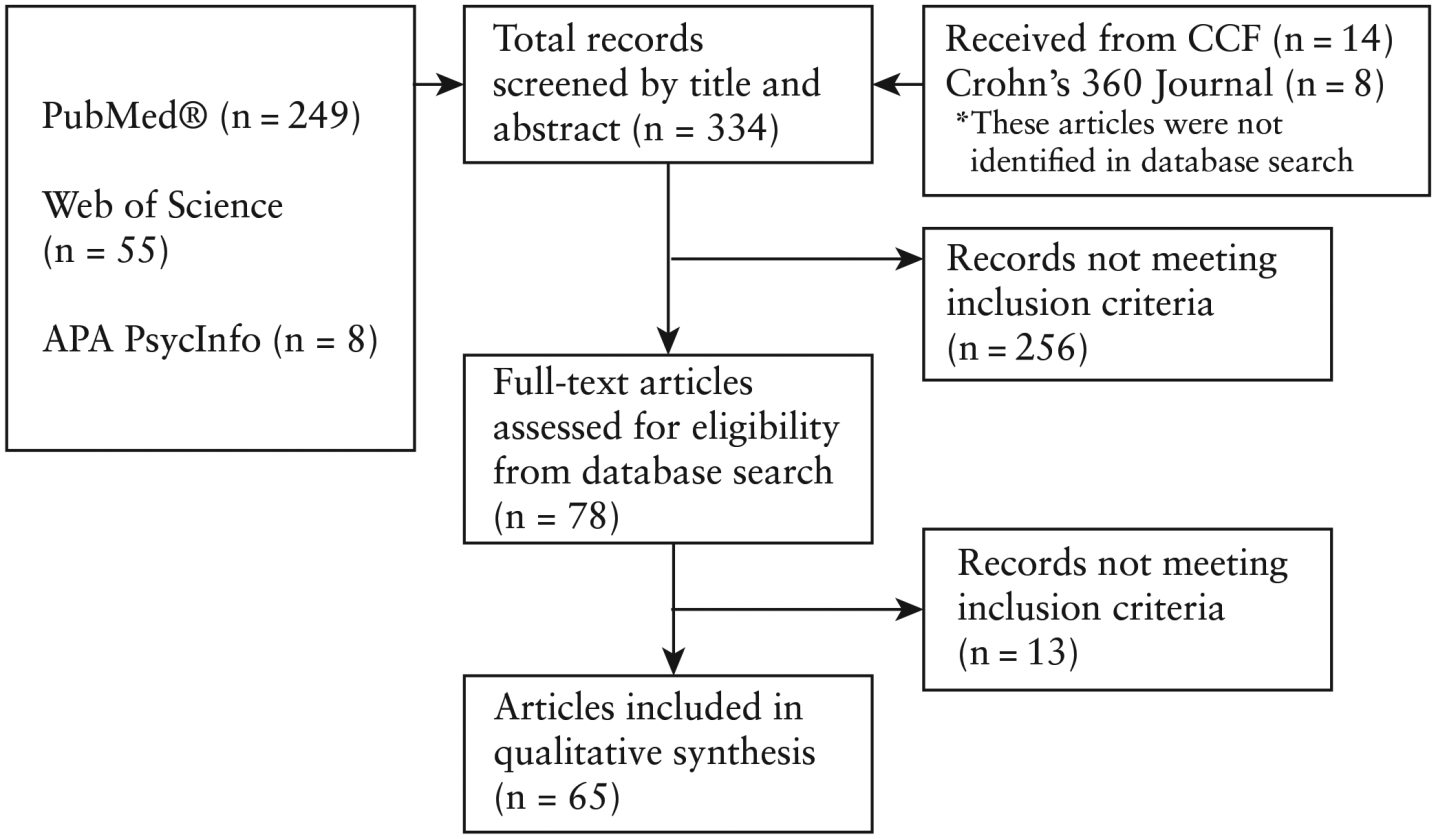
Flowchart for article search, screening, retrieval, and review. CCF, Crohn’s and Colitis Foundation.

The electronic search identified 334 potentially eligible studies for screening after the addition of the manual searches (Figure 2). Seventy-eight articles were considered for full evaluation. Sixty-five articles met the inclusion criteria for data extraction. Forty-eight studies included factors relating to screening, 10 studies included factors relating to diagnosis, and 54 studies included factors relating to treatment.

Multiple studies included more than 1 category among screening, diagnosis, and treatment factors. Four additional approaches as part of the provision of care were identified and included.

### Consensus Statements

Using input from the literature scan and their own expertise and experience, the expert panel developed 6 consensus statements (Table 1) that serve to provide IBD healthcare professionals with evidence-based suggestions for working with patients who may need additional resources or treatment for depression, anxiety, or both. Although these statements provide a starting point for IBD healthcare professionals to work with their patients, they are not intended to be an exhaustive set of recommendations for every scenario. Additional discussion is needed within individual IBD care teams to identify best practices specific to their teams, resources, context, and patients.

**Table 1.** Final consensus statements

Statement
1. Initiate and maintain conversations regarding mental and emotional well-being with patients to provide information and support regarding the bidirectional relationship between and co-occurrence of anxiety and depression and IBD disease course.
2. Routinely screen all IBD patients for mental and emotional well-being, including anxiety and depression.
3. Seek out educational opportunities and training on how to effectively respond to patients who disclose or screen positive for anxiety and depression symptoms, including suicidal ideation.
4. Demonstrate competence in the ability to have an informed conversation with patients regarding various evidence-based treatment approaches for anxiety and depression and effects of treatment approaches when offering resources and referrals.
5. Identify, maintain, and share a list/database of resources (eg, mental health providers, websites, apps) that patients can access to manage and treat their anxiety and depression symptoms.
6. Encourage patients to pursue evidence-based treatment approaches for symptoms of their anxiety and depression.

Abbreviation: IBD, inflammatory bowel disease.

Consensus Statement 1. Initiate and maintain conversations regarding mental and emotional well-being with patients to provide information and support regarding the bidirectional relationship between and co-occurrence of anxiety and depression and IBD disease course.

Panel members sought to emphasize the importance of IBD healthcare professionals normalizing patient-provider conversations surrounding mental health and emotional well-being, considering the bidirectional effects between anxiety and depression symptoms and the course of IBD. Anxiety and depression may increase the risk for development of Crohn’s disease and ulcerative colitis by driving inflammation and exacerbating IBD disease symptoms, as well as by increasing the likelihood of recurrence; simultaneously, the onset of depression can affect one’s quality of life, self-management behaviors, and adherence to treatment.^10,20,21^ Thus, IBD healthcare professionals are strongly encouraged to raise these issues, using patient-friendly language to explain the connection between IBD disease activity and anxiety and depression to reduce mental health-related stigma, for instance, “It is normal to feel sad and anxious as you live with inflammatory bowel disease” to initiate a more detailed discussion. Subsequently, IBD healthcare professionals can encourage and facilitate patient referrals when psychological intervention or treatment is needed.^21^

Consensus Statement 2. Routinely screen all IBD patients for mental and emotional well-being, including symptoms of anxiety and depression.

Screening for anxiety and depression—through informal discussions and/or validated formal screening questionnaires—should be conducted as part of routine care for IBD patients, as psychological symptoms are often present throughout the disease course in IBD patients.^22^ Only an IBD care team clinician (eg, physician, advanced practice provider, dietitian, psychologist, social worker) should conduct screening. Initiating brief conversations at every appointment as a part of the review of symptoms may help normalize conversations surrounding mental health; asking a simple, open-ended question such as, “How have you been coping/managing lately?” may help start the discussion. It is suggested that formal screening tools should be administered at least annually or as clinically indicated—these can be administered by office staff or any member of the IBD care team, but results should be reviewed with the patient by their primary IBD healthcare professional. This is recommended in the event that screening suggests that a patient is experiencing extreme anxiety or depression or in cases in which suicidality is indicated.

To extend accessibility to diverse populations, healthcare professionals should consider administering screenings tools that are available for both English- and non–English-speaking patients. Table 2 provides a list of screening tools that were reported to be commonly used by IBD clinicians and mental health professionals. Regardless of screening method, healthcare professionals should always aim to provide culturally sensitive care and use inclusive language during screening to maintain a safe and supportive environment for patients.

**Table 2.** Screening tools

Screening tool name	Key characteristics					
	Patient age (y)	Cost per test	Who can administer the tool?	Delivery in person or online	Time to administer screener (min)	Other languages available (besides English)
Both anxiety and depression						
Patient-Reported Outcomes Measurement Information System (PROMIS)^23,24^	Adult and pediatric patients	Free (by paper in English)	Self-report	Both	~3	Spanish
Depression						
Beck Depression Inventory^25,26^	13-80	>$100	Self-administered or administered verbally by a trained administrator	Both	5	Spanish
Child Depression Inventory^27,28^	7-17	>$300	Self-report	Both	5-15	Spanish
Moods and Feelings Questionnaire^29–31^	6-19	Free	Formal training is not required to administer	In person	10	Over 10 languages
Patient Health Questionnaire (PHQ-2 and PHQ-9)^32–34^	12 and older	Free	Self-administered or clinician-administered	Both	2-5	Over 20 languages
Anxiety						
Beck Anxiety Inventory^35,36^	17-80	>$100	Self-administered or administered verbally by a trained administrator	Both	5-10	Spanish
General Anxiety Disorder-7 ^32,37,38^	12 and older	Free	Self-administered or clinician administered	Both	2-5	Over 10 languages

Screening results should be evaluated in real time by a professional. Some tools may include sensitive questions, such as those assessing suicidality (eg, Beck Depression Inventory, Child Depression Inventory, PHQ-2, PHQ-9); these questions should only be administered by a trained professional and interpreted immediately, or omitted if a professional is unable to promptly review and provide support.

Abbreviation: PHQ, Patient Health Questionnaire.

Consensus Statement 3. Seek out educational opportunities and training on how to effectively respond to patients who disclose or screen positive for anxiety and depression symptoms, including suicidal ideation.

IBD professionals should explore learning opportunities to address the gap in physician awareness and training with regard to managing IBD patients with comorbid anxiety and depression.^39^ For example, staff training on mental health management for all members of the IBD care team helps ensure successful anxiety and depression screening implementation, so that each provider understands their role in the process, practices the necessary skills, and feels more confident in conducting screening and follow-up. If screening indicates anxiety or depression symptoms are present, IBD healthcare professionals should be prepared to provide resources to patients that offer guidance on how to find and access additional mental health support and care, possible treatment options, and resources that can be accessed in their community. These resources should be readily available to ensure that patients can receive prompt access to services and timely care.^40,41^ The Crohn’s and Colitis Foundation website (https://www.crohnscolitisfoundation.org/science-and-professionals/education-resources) is a starting point that provides healthcare professionals with a variety of educational modules, resources, and training.^42^ In the event of a positive patient screening result for suicide risk, healthcare professionals should be prepared to follow up immediately and provide appropriate care. Thus, providers are strongly encouraged to participate in suicide prevention trainings to ensure that they are equipped with the necessary skills to provide support.

Consensus Statement 4. Demonstrate competence in having an informed conversation with patients regarding various evidence-based treatment approaches for anxiety and depression and the effects of these treatment approaches when offering resources and referrals.

There are a wide variety of treatment approaches available to patients dealing with anxiety and depression. Although IBD healthcare professionals are not expected to manage the anxiety and depression of their patients, they are strongly encouraged to develop and demonstrate competence of these treatment approaches so that they are prepared to have an informed conversation with their patients when needed. Treatments range from self-care practices and strategies to reduce stress to psychosocial interventions with trained therapists and psychopharmacological interventions. Table 3 provides examples of these treatment approaches as a starting point for familiarization. Self-administered interventions, for example, are those that can be recommended or provided by any member of the IBD care team and may be effective if a patient is experiencing mild anxiety or depressive symptoms. They can also be combined with other treatments for more severe anxiety and depression. Psychosocial approaches are provided by trained mental health professionals and can often be delivered in person or through virtual/telehealth meetings. Both modalities can also be paired with psychopharmacological approaches. Because patient preferences for treatment are critical to treatment adherence, IBD healthcare professionals are encouraged to become familiar with various treatment approaches so that the provider and patient can work collaboratively to find the appropriate patient supports. IBD healthcare professionals should also be knowledgeable of the effects of certain treatment approaches when offering resources or referrals. For example, it is important to inform patients that corticosteroid use may contribute to psychological morbidity when offering this class of medications to reduce inflammation.^50^

**Table 3.** Sample treatment approaches

Sample treatment approaches	Brief description/reason for inclusion	Provision by IBD professionals
Self-administered interventions		
Diaphragmatic breathing	Involves breathing deeply and expanding the lungs into the diaphragm^43^. May decrease stress as measured by physiologic biomarkers, as well as psychological self-report tools.^3^	IBD professionals are able to recommend and provide instruction on this intervention.
Yoga	Offers physical and mental health benefits for people of all ages, including beneficial effects on health-related quality of life and anxiety.^44^	IBD professionals are able to recommend and provide resources on this intervention.
Mindfulness-based stress reduction	An effective method for reducing physical and psychological suffering while building resilience, balance, and peace of mind.	IBD professionals are able to recommend and provide resources on this intervention.
Psychosocial interventions		
CBT	CBT has been demonstrated to be effective for treatment of depression and anxiety disorders.^41,45,46^	IBD professionals are able to recommend this intervention but should refer to a mental health professional for its provision.
Primary and secondary control enhancement therapy	A disease-specific CBT protocol for youth with depression.	IBD professionals are able to recommend this intervention but should refer to a mental health professional for its provision.
Behavioral activation therapy	A type of CBT that serves as a simple psychological treatment for depression and is widely accepted.^47^	IBD professionals are able to recommend this intervention but should refer to a mental health professional for its provision.
Pharmacological interventions		
ADM	Suggested ADMs include SSRIs, TCAs, and SNRIs. SSRIs and SNRIs can also be used as a first-choice treatment for generalized anxiety.^48^	IBD professionals are able to recommend and provide this intervention but may also wish to engage a psychiatrist.
Anti-TNF-α therapy	A type of treatment in which medication reduces active inflammatory cells in the intestinal tissue. Evidence shows that anti-inflammatory treatments show promising effects on improving depressive symptoms in the general population.^49^	IBD professionals are able to recommend and provide this intervention.

Abbreviations: ADM, antidepressant medication; CBT, cognitive behavioral therapy; IBD, inflammatory bowel disease; SNRI, serotonin and norepinephrine reuptake inhibitor; SSRI, selective serotonin reuptake inhibitor; TCA, tricyclic antidepressant; TNF-α, tumor necrosis factor α.

Consensus Statement 5. Identify, maintain, and share a list or database of resources (eg, mental health providers, websites, apps) that patients can access to manage and treat their anxiety and depression symptoms.

IBD healthcare professionals are strongly encouraged to develop and maintain a list of mental health resources that can be provided to patients with depression and anxiety symptoms or diagnosis. Every community or practice will not have access to the same resources. Thus, it may be important not only to identify local resources, but other resources that may be available through telehealth, websites, or apps. For example, the Crohn’s and Colitis Foundation Depression and Anxiety webpage (www.crohnscolitisfoundation.org/mental-health/depression-anxiety) provides patients with information, resources, and references^51^ on these important issues. When possible, IBD professionals should seek to develop or participate in a multidisciplinary team comprising providers focusing on the medical management of IBD activity and those focusing on the treatment of anxiety and depression symptoms (eg, psychologists, social workers). Involvement in a multidisciplinary team can facilitate the development and maintenance of a resource database and serve to establish a referral network in which patients can access additional mental health resources.

Consensus Statement 6. Encourage patients to pursue evidence-based treatment approaches for symptoms of their anxiety and depression.

The literature is clear that many patients with IBD have a higher risk of experiencing symptoms of anxiety and depression^3,4^ and will need support to normalize and manage those symptoms. IBD healthcare professionals play a critical role in providing both support and resources for these patients. Because of the stigma that often exists around mental health,^52^ patients may need encouragement to pursue evidence-based treatments for their anxiety and depression. Establishing a process to link patients to appropriate referrals and resources is critical. Unfortunately, delays between screening and treatment may occur due to several factors, including limited availability of mental health resources in a community, long wait times to access a mental health provider, patient insurance coverage and approvals, or patient ability to travel to another provider. In these situations, it is important for IBD healthcare professionals to make patients aware of mental health resources available to them. While awaiting professional mental healthcare, patients can also be instructed on how to implement some of the self-administered interventions and how to access psychosocial care through apps and telehealth resources. IBD professionals are also encouraged to familiarize themselves with 1 or 2 antidepressant medications. Selective serotonin reuptake inhibitors and tricyclic antidepressants are well established in the treatment of anxiety and depression, though limited studies have been conducted on IBD patients.^53^ In certain cases, the IBD healthcare professionals who are properly trained on these medications may want to prescribe and manage these medications for their patients.

## Discussion

Mental healthcare and support for IBD patients is critical. The statements included in this article represent a small set of practical considerations for IBD healthcare professionals. The statements address key issues around provider awareness, knowledge and behaviors, screening and treatment resources, and patient education. These statements are not intended to be comprehensive guidance, but rather are intended to be a summary of important issues providers need to consider as they provide patient care. Although many of these statements are supported by strong evidence within the IBD literature, some are supported by broader healthcare delivery best practices, such as using screening tools in languages other than English for patient comfort as needed, using patient-friendly and inclusive language when discussing mental health and IBD co-occurrence, and considering patient preferences when discussing treatment options. We chose to retain these important considerations because, although they may not be explicitly discussed within the current IBD literature, a strong evidence base does exist within broader best practices.
