# Correction to: Consensus Statement on Managing Anxiety and Depression in Individuals with Inflammatory Bowel Disease

**DOI:** 10.1093/ibd/izae243

**Published:** 2024-09-25

This is a correction to: Laurie Hinnant, Nicholas Rios Villacorta, Eliza Chen, Donna Bacchus, Jennifer Dotson, Ruby Greywoode, Laurie Keefer, Stephen Lupe, Leah Maggs, Garrett Meek, Eva Szigethy, Kathryn Tomasino, Orna G Ehrlich, Sylvia Ehle, Consensus Statement on Managing Anxiety and Depression in Individuals with Inflammatory Bowel Disease, *Inflammatory Bowel Diseases*, 2024; https://doi.org/10.1093/ibd/izae151

In the originally published version of the manuscript there was a typographical error in the first name of the 12th author. This has now been emended in the article to read: “Kathryn Tomasino”.

